# Rank of Surgeons in the Navy

**Published:** 1852-07

**Authors:** 


*Rank of Surgeons in the Navy.*—The claim of the Surgeons of the navy to an assimilated rank, has been for several years before the profession and the country; but justice to our brethren connected with that arm of our national defence has not yet been obtained. Efforts should not, and it is to hoped will not be abandoned, until the end be attained; and perseverance will, we doubt not, sooner or later, be crowned with success. This subject was brought up at the late meeting of the Am. Med. Association, and referred to a committee, who submitted, by their chairman, Dr. Jackson, of Pennsylvania, the following report, which will be read with interest:

The committee to whom the resolutions relative to the rank of the medical officers of the United States navy were referred, respectfully report:

That the objects sought to be obtained, in respect to rank, by the medical officers of the navy, and the course they have pursued, were approved by this association at the meetings held in Baltimore 1848, in Cincinnati, Ohio, 1850, and in Charleston, South Carolina, 1851.

The attention of the association has again been directed to this subject by Surgeon Ninian Pinkney, of the navy, who has read a memorial he contemplates presenting to congress, and a bill providing by legislative enactment for the definite settlement of this unnecessarily debated question.

This association can have no hesitation in reaffirming its former opinions and action, and of approving the memorial of Surgeon Pinkney so far as it sustains the views of the navy medical officers, as also the provision contained in the bill which he has drafted for the adjustment of this controversy, calculated to disturb the harmony of the officers associated in separate departments of the same service, and whose united action is indispensable to the perfect fulfillment of their respective duties.

It is difficult to understand the opposition of the naval ship officers to the institution of military rank and grade for the navy medical officers. Rank and grade are things in themselves of no value—that philosophers may despise—but it is the universal custom of mankind to employ them as symbols of ideas expressive of honor and respect for individuals or stations. They do not necessarily, and need not confer command, or be connected with other than moral power and influence. The naval ship officers comprehend fully the value of rank and grade and forms of ceremony on the minds of the crews, who are for the most part uneducated, whom they command. They evince on this very subject great pertinacity, and it may also be said jealousy, in the opposition they make to the conferring of rank and grade unassociated with command or power, as marks only of honor and respect, on the naval medical officers. The government some years past, by the appointment of a navy and army examining board, elevated the standard of education for their navy and army medical officers to the highest point, not merely for professional knowledge, but on subjects of general information. It is a higher standard than that of any of our medical schools.

The navy department, acting in uniformity to this requisition of higher attainments in the medical officer, in the year 1847 conferred on him assimilated rank and grade, denoting solely the honor and respect due to him and his position, bestowing no power that could interfere with the command and proper duties of the naval ship officers.

So far as this association has information, this system worked well—did not conflict with any duties connected with the command of the ship, and that no valid reason existed for its alteration. It was not permitted to remain undisturbed. It was subjected to a revision by a board of officers, in which the medical navy officers had not a single representative. The result has been a report that degrades the navy medical officers from their former rank, and establishes a lower one, and without assigning a reason for this course, or the advantages it possesses over that which had been previously established by the navy department. What renders this proceeding the more invidious is, that the army board, appointed at the time with the navy board and for similar enquiries, have retained the rank and grade of the army medical officers as previously established, which was the same as that of the navy medical officers under the regulation of 1847.

As a consequence of this different action of the boards of navy and army officers, this incongruity, if adopted, will be introduced into the military code of the United States—that the navy medical officers will hold a lower grade and rank than the army medical officers, and a consequent implication of the inferiority of the one to the other.

In view of the above considerations, the committee submit to the association the following resolutions as substitutes for those referred to them:

1. *Resolved*, That the American Medical Association, representing the medical profession of the United States, reaffirm the resolutions passed at the meetings held in Baltimore in 1848, in Cincinnati in 1850, and in Charleston, South Carolina, in 1851, by pressing their approbation and support of the establishment of the assimilated rank conferred on the navy medical officers by the regulation of the navy department in 1847.

2. That this association is not aware of any disadvantage attending on the regulation of 1847; that they can perceive no just cause for its alteration, and disapprove of the change proposed.

3. That it is the opinion of this association that it would be for the interest of the naval service that this question should be settled definitively during the present session of congress, and, if conformable with the usages of the military service, by legislative enactment, to which request they respectfully invite the attention of the honorable senate and house of representatives.

SAM'L JACKSON.
CHRIS. C. COX.

On motion of Dr. Corbin, of Va., the report was received and adopted, and the secretaries were instructed to forward a certified copy of the same to the presiding officers of both houses of congress, and also to the secretary of the navy.

